# Crime Light Imaging (CLI): A Novel Sensor for Stand-Off Detection and Localization of Forensic Traces

**DOI:** 10.3390/s23187736

**Published:** 2023-09-07

**Authors:** Andrea Chiuri, Roberto Chirico, Federico Angelini, Fabrizio Andreoli, Ivano Menicucci, Marcello Nuvoli, Cristina Cano-Trujillo, Gemma Montalvo, Violeta Lazic

**Affiliations:** 1 Italian National Agency for New Technologies, Energy and Sustainable Economic Development (ENEA), Laboratory FSN-TECFIS-DIM, Via Enrico Fermi 45, 00044 Frascati, Italy; andrea.chiuri@enea.it (A.C.); roberto.chirico@enea.it (R.C.); marcello.nuvoli@enea.it (M.N.); 2 Italian National Agency for New Technologies, Energy and Sustainable Economic Development (ENEA), Laboratory FSN-FUSEN-TEN, Via Enrico Fermi 45, 00044 Frascati, Italy; 3 Universidad de Alcalá, Departamento de Química Analítica, Química Física e Ingeniería Química, Facultad de Farmacia, 28871 Alcalá de Henares, Spain; 4 Universidad de Alcalá, Instituto Universitario de Investigación en Ciencias Policiales, Calle Libreros 27, 28801 Alcalá de Henares, Spain

**Keywords:** crime scene, imaging, stand-off, forensic lights, latent traces, fingerprints, blood, semen, 3D reconstruction, scanning

## Abstract

Stand-off detection of latent traces avoids the scene alteration that might occur during close inspection by handheld forensic lights. Here, we describe a novel sensor, named Crime Light Imaging (CLI), designed to perform high-resolution photography of targets at a distance of 2–10 m and to visualize some common latent traces. CLI is based on four high-power illumination LEDs and one color CMOS camera with a motorized objective plus frontal filters; the LEDs and camera could be synchronized to obtain short-exposure images weakly dependent on the ambient light. The sensor is integrated into a motorized platform, providing the target scanning and necessary information for 3D scene reconstruction. The whole system is portable and equipped with a user-friendly interface. The preliminary tests of CLI on fingerprints at distance of 7 m showed an excellent image resolution and drastic contrast enhancement under green LED light. At the same distance, a small (1 µL) blood droplet on black tissue was captured by CLI under NIR LED, while a trace from 15 µL semen on white cotton became visible under UV LED illumination. These results represent the first demonstration of true stand-off photography of latent traces, thus opening the way for a completely new approach in crime scene forensic examination.

## 1. Introduction

The investigation of a crime scene is a complex process that involves spatial reconstruction and accurate localizations of evidence, and then collection, identification and documenting of the evidence, as well as the preservation of some samples for further analysis in laboratories (i.e., DNA extraction). This investigation process should also preserve the scene with minimal contamination and disturbance, and it should be conducted in an expeditious and methodical way following the best practice [1]. Technological developments might importantly help the investigators where, for example, in outdoor scenarios, the use of drones equipped with cameras [2] or other sensor [3,4] supplies may rapidly capture crucial data without altering the evidence. Both in outdoor and indoor situations, 3D crime scene reconstruction is of fundamental importance [5,6], where it is possible to exploit photogrammetry or to use modern 3D scanners that combine photography and laser distance measurements from the observation point or employ time-of-flight cameras. Whereas 3D scanners offer a relatively rapid and more comprehensive view of geometry of the whole scene, classical digital photography provides better information about small details, which can be then localized on 3D pictures by applying photogrammetry [7,8].

Direct photography of the details of a crime scene might not be sufficient itself, as some forensically important traces (e.g., bodily fluid stains, footprints, fingerprints) can occur on substrates which color or transparency prevents their straightforward detection. In such cases, the so-called latent traces can be observed using adequate alternate light sources (ALSs) and/or specific chemical agents. The ALSs have a role in illuminating samples at certain wavelengths in order to make possible latent traces visible [9,10], mainly thanks to the following phenomena [11]: (1) the evidence absorbs the impinging light while the substrate does not absorb it (or vice versa); (2) the evidence produces light scattering while the substrate reflects or absorbs the incident light, or it is transparent for the same reason; (3) the evidence is fluorescing, and it can be visualized by a suitable configuration of the detector. Stains of some biological fluids, like semen, saliva and urine, produce fluorescence under proper ALS illumination [4,12]. On the other hand, there are various disposable products, like fluorescent powders, sprays for latent prints and chemicals for bodily fluids, such as luminol, that highlight the latent trace without or with use of ALSs [4]. Both the ALSs and the specific chemicals are important for the detection of a great variety of latent traces that can be found on the crime scene; these techniques are portable, cheap, easy to use and can be operated in combination with chemical screening tests [13]. The visualization of latent traces does not represent sample identification but indicates possible evidence; locating the traces also helps successive sample retrieval, classification and documentation [14].

Due to a large variability of possible latent traces and substrates on crime scenes, the ALSs should include a set of illumination sources from the UV to NIR spectral range in order to cover, for example, the detection of untreated or treated fingerprints [15,16,17], fibers [18,19], blood [20], body excretions [21,22,23,24], drugs [25], bruises [26], gunshot residues [27,28,29], bones [30,31] and papers and inks [28,32,33]. By combining a proper illumination source and wavelength filtering of the signal in front of a photo camera, the capability to detect latent traces on a larger number of substrates can be achieved [12,24].

Although the methods for the close detection of forensic traces are already well established, there is a technological difficulty to achieve the same results at distances of several meters or more. The first problem here is related to the angular spreading of the illumination from common ALSs, which reduces the radiation density on the target with its distance, therefore limiting the working range. On the other hand, collimated laser beams might propagate at long distances, maintaining a high radiation density on the target. However, if excluding relatively large and expensive tunable laser sources, the laser emissions at single narrow wavelengths limit the possibilities for detecting a wide range of latent traces, in contrast to commercially available ALS kits for close inspection that contain a number of light sources emitting from UV to NIR [34]. Another problem arising in the stand-off photography of latent traces is due to ambient light that, particularly in sunny day conditions, might completely mask the target illumination by a directed laser or ALS light. In the case of stand-off spectroscopic measurements, for example, by the Raman technique [35], the signal masking by the background light can be minimized using short illumination pulses (in order of ns or less) and synchronized gated detection. Such measurements employ fast detectors, like Intensified Charge Coupled Devices (ICCDs) that are very costly and produce only a monochromatic image. 

One of very few existing prototype instruments for the stand-off detection of latent traces was described in [36]. This instrument is based on target illumination by an assembly of continuously emitting blue LEDs (420–470 nm) and a commercial 3D camera. The testing was performed in a laboratory at distances up to 3 m, obtaining detection rates of semen and saliva stains that were strongly dependent on the substrate color and sample volume (5–250 µL). In the successive work, the same authors employed three illumination sources with narrow wavelengths at 405 nm, 445 nm and 532 nm, extending the study to blood droplets and fingermarks [37]. The achieved detection rates for blood stains were between 0% and 100%, depending on substrate’s color and the exposure time (varied between 30 s and 12 min), and the image quality was also affected by ambient light. In case of semen or saliva stains in volume of 250 µL placed on various substrates, the final detection rate was of 100% thanks to the case-by-case optimization of the illumination wavelength and exposure time. 

After reviewing the existing literature, guidelines for practitioners and available commercial or prototype instruments, we believe that the stand-off photographic detection of latent traces still remains an important target to reach, particularly due to the signal loss when increasing the working distance, the difficulties of combining light sources with very different wavelengths into a single illumination spot and the influence of ambient light on the measurements.

In this work, we present a novel stand-off sensor called Crime Light Imaging (CLI) that has been projected to operate for target distances between 2 m and 10 m. This device is based on a High-Definition (HD) color camera and on four high-power LEDs emitting from near-UV to NIR spectral region. The illumination LEDs were accurately selected to cover a wide range of possible latent traces without or with chemical pretreatment. The CLI was also equipped with a laser distance meter for guiding the sensor focusing and providing the input for 3D scene reconstruction. Although the CLI is an operative stand-alone device, we integrated this instrument into a mobile positioning system (tripod with motorized platform and panoramic camera) with the aim to reach a fully automated 3D reconstruction of the scene or to provide exact locations of evidence on a large scene reconstructed by fast commercial systems in the future. The second operative approach is already in use inside the Real-time on-site forenSic tracE qualificatioN (RISEN) project, dealing with augmented crime scene investigation through an interactive 3D model; this model includes the exact positions and labels of the traces detected by various contactless sensors [38].

## 2. CLI Sensor Development 

In the following section, we describe the CLI instrument and its functionalities, while the instrument performances measured on a selected set of samples, namely fingerprints, blood stains on black cotton and semen stains on white cotton, are reported in the Results section. In the selection of the representative samples/substrates, we had to consider that crime scenes are always complex. Bodily fluids and fingerprints are some of the most important evidence at a crime scene because they make it possible to extract DNA and attribute the traces to a person (not necessarily the suspect/s). Visually, they are not easy to detect, and some environmental interference might occur, such as, for example, false positives for substances resembling stains of biological fluids. In particular, blood on black and semen on white substrates are difficult to visualize under ambient light and during close examination; consequently, their detection as small stain spots and at a distance of several meters becomes an extremely challenging task.

### 2.1. Input Requirements and Main Hardware Components

Basing on the inputs from Law Enforcement Agencies involved in the RISEN project [38], our final design for realization of the CLI instrument was shaped to comply with the following main requirements:

- The working distance should enclose the range between 2 m and 10 m, where the shorter working distances are expected in indoor scenarios and the longer ones might be necessary in outdoor environments.
- The target illumination by ALSs should allow the detection of a wide range of forensic traces; this and the previous requirement were translated into a choice of four high-power LEDs emitting from near-UV to near-NIR wavelengths.
- The photographic image resolution should be better than 100 µm and thus sufficient to visualize fingerprints, hair and fibers.
- The instrument should make it possible to localize the traces on a 3D scene and should have auto-focusing capability; this means that it is necessary to employ distance measurements and motorized stages.
- The instrument functioning should have a relatively low power consumption, allowing for one hour battery operation.
- The sensor should be transportable, and its head must be sufficiently lightweight (<12 kg) to be mounted on a scanning system.
- The instrument deployment and use are expected to be relatively fast and simple, with a user-friendly graphical interface.

The main functional hardware components of here presented CLI sensor are:

1. Illumination system
2. Detection system
3. Laser pointer/distancemeter
4. Electronic control
5. Mobile positioning system (MPS)

**Figure 1:**
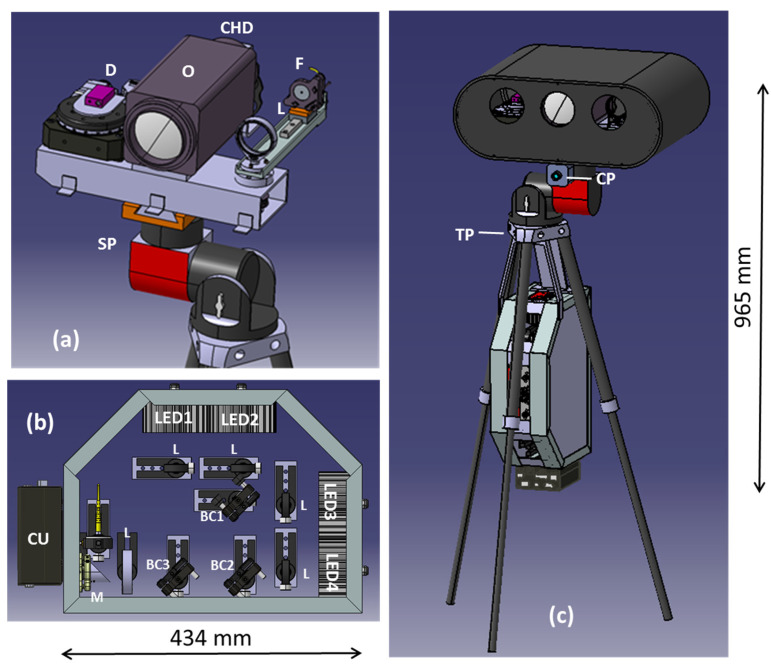
(**a**) Sensor head mounted on the scanning platform (SP). (**b**) Instrument box. (**c**) CLI sensor integrated into the MPS containing tripod, SP and panoramic camera (CP); on the lower side of the tripod plate (TP), the instrument box was fixed via two (frontal) brackets and one thick (back) holder.

The details about each of the main components are given in following sections. In order to facilitate the instrument deployment by a single operator and to minimize the weight of the components mounted on the positioning platform, the hardware was split into two parts, namely the sensor head (Figure 1a) and the instrument box (Figure 1b). The central part of the first unit contained a color HD (CHD) camera with objective (O), while the distancemeter (D) and the unit containing fiber (F) attachment plus optics (L) for the illumination light were symmetrically shifted from the center and mounted on rotating cylinders. These cylinders were simultaneously moved by a home-built scissor-like mechanism, herein called the convergence actuator, based on a rotating motorized stage (Thorlabs HDR50/M with controller BSC201).

The sensor head could be rapidly fixed on the scanning platform of the MPS by means of two fast clumps. The head was connected to the instrument box via a 2 m-long umbilical enclosing electrical and communication cables plus an optical fiber for transmitting the illumination light. The instrument box contained four LED sources with the corresponding optical elements that included lenses (L), beam combiners (BC1–3) and one off-axis parabolic mirror (M). A control unit (CU) powered by 24 VDC or 220 VAC was fixed on the box externally in order to facilitate connections of the cables. The whole box was attached on lower side of the tripod’s plate by three holders, in a way to have equilibrated weight distribution among the tripod’s legs (Figure 1c).

#### 2.1.1. Illumination System

The choice of the illumination LEDs for CLI was based on a need to have high-power pulsed light sources emitting at wavelengths that allow the detection of a wide range of possible latent traces, without or with chemical pretreatment. The pulsed illumination, here down to ~10 µs duration, made it possible to increase the LED’s current, i.e., radiation density on the target compared to the continuous operating mode. Furthermore, the pulsed LED’s emission, when synchronized with a short exposure (~10 µs) photographing camera, made it possible to minimize the impact of ambient light on the measurements. In such conditions, the luminosity of the target image can be enhanced by acquiring more frames and applying successively a post-processing that sums the intensities of each pixel in the frame sequence.

**Figure 2:**
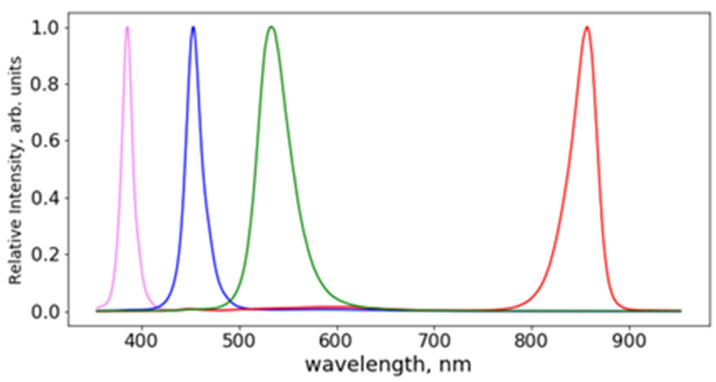
Spectral emissions from the LED sources employed in the CLI sensor.

The chosen LEDs were EffiLux EFFI-SHARP-PWR-CM-xxx-STR, where xxx denotes the wavelength of the spectral peak emission. The LEDs were driven by the dedicated controllers (Smartek HPSC4) and emitted at peak wavelengths of 385 nm, 455 nm, 520 nm and 850 nm. The LED spectra measured by a compact spectrometer (Ocean Insight QEPRO-FL) with resolution of 1.5 nm are shown in Figure 2. The spectral widths, calculated as Full Width Half Maximum (FWHM), were between 20 nm for the UV LED and 50 nm for the green-emitting source. These relatively large bandwidths do not represent an issue for photography here, differently from some spectroscopic techniques (e.g., IR absorption, Raman spectroscopy) that require a narrowband light excitation. According to the literature, the types of traces expected to be optimally detected by each of the selected LED wavelength, as well as the corresponding physical processes (examination form) mainly responsible for their distinguishing, are listed in Table 1.

The maximum measured LED powers in continuous operation mode, with a current of 1.0 A, are 1 mW for UV-VIS light or 2 mW for the NIR source. However, if the LED is operated in a pulsed mode and with a small duty cycle D, defined as ratio of the pulse duration τ to period T, the pumping current could be increased. According to the manufacturer’s specifications, for example, with D = 0.1 and τ ≤ 10 ms, the maximum current is 1.5 A, while for D = 0.0004 and τ ≤ 10 µs, the current could reach 3.5 A. This means that the pulsed LEDs can provide much more intense light than in continuous mode, which is important for efficient imaging.

Inside the instrument box (see Figure 1b), we coaxially combined the previously collimated lights from four LEDs. The light is then focused onto a large core optical large fiber by a lens and off-axis parabolic mirror. The optical fiber is 2 m long, and it carries the light to the mobile sensor head (see Figure 1a), where its output passes through an achromatic lens before being directed to the target. The fiber’s holder is mounted on a motorized slit (Micronix VT-50L-11310) in order to vary size of the illumination spot and match it as much as possible with to the size of the object to be photographed, also taking into account the measured working distance and desired image resolution. The minimum illumination spot on the target is about 50 mm diameter. The LEDs are operated one at a time.

#### 2.1.2. Detection System

The detection system consists of an HD color camera, motorized zoom lens and motorized wheel with filters. The camera chosen for the CLI instrument (Baumer VCXU-50C) is based on a 2/3″ CMOS sensor (SONY IMX250) with 2448 × 2048 pixels and with a minimum integration time of 1 µs. The camera, controlled via a USB 3.0 interface, can be externally triggered, and its capability to perform a short exposure signal acquisition synchronized with the pulsed LED light is extremely important for minimizing the influence of ambient light on the acquired frames. The short-exposure multiple frames can also be summed to increase the image quality.

**Figure 3:**
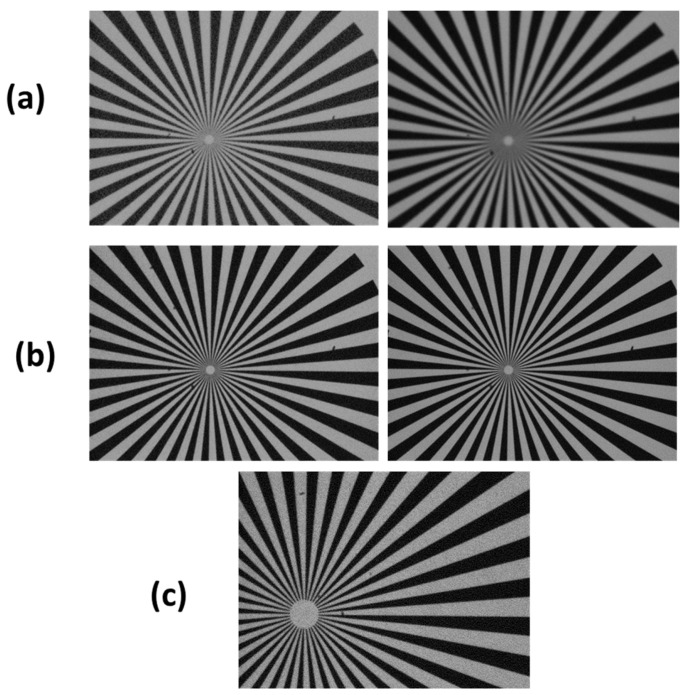
Photographs of the testing target taken at distance of 2 m (**a**) and 2.8 m (**b**) with the minimum (left column) and optimized (right column) iris aperture, where the object size was ≈120 mm large; (**c**) picture acquired at distance of 2.8 m with the maximum objective’s zooming.

The objective in front of the camera (Fujinon FH32x15.6SR4A-CV1) has a motorized variable iris and focal length between 15.6 mm and 500 mm. Its control via RS-232 interface allows the user to adapt remotely the lens parameters to the specific object, distance and illumination conditions. The optimum focusing range for the objective is from 3 m to infinity; however, it is still possible to take pictures at distance of 2 m, although with some loss of image sharpness below 2.8 m (Figure 3).

When dealing with photography of trace evidence (e.g., fingerprints, hair) it is necessary to achieve a high spatial resolution, typically better than 100 µm. For the selected combination of the sensor and objective, the maximum theoretical spatial resolution, calculated for the objective’s focal length of 500 mm (the maximum zoom). is reported in Table 2, and it remains well below 100 µm for the target distances up to 10 m. The minimum LED spot size on the target is of about 50 mm diameter, which is slightly larger than the minimum object’s size that can be photographed at a distance of 3 m. Thanks to the variable distance between the collimating lens and fiber tip transmitting the LED light, the illumination spot can be enlarged, for example, to 162 mm diameter, corresponding to the minimum area that can be photographed at distance of 10 m. By reducing the objective’s focal length and thus enlarging the object area, a faster scanning of the crime scene can be achieved at the expense of details, i.e., spatial resolution.

The possibility to take photographs only of the backscattered or fluorescent light is provided by the filters mounted on a six-hole motorized wheel (Thorlabs FW102C) inserted between the objective and camera. The wheel currently houses five filters, while one aperture remains free and plays the role of a neutral filter. 

#### 2.1.3. Laser Pointer/Distancemeter 

Measurements of the target distance are performed by a compact high-frequency laser distance sensor (M88B JRT) designed to operate in a range of 0.03–60 m with an accuracy of ±1 mm. This device is powered and controlled via USB cable. The laser’s peak emission is at 650 nm. The laser spot is brought to the center of the area to be photographed by the convergence actuator; this assists the operator to tag accurately the central point of interest. For the selected area to be photographed, the first image is acquired in presence of the pointing beam with the aim to facilitate the localization of this area in a large scene provided by the external 3D scanner. The successive photograms of the same area, performed without or with LED’s illumination, are captured after turning off the pointing beam.

#### 2.1.4. Electronic Control

**Figure 4:**
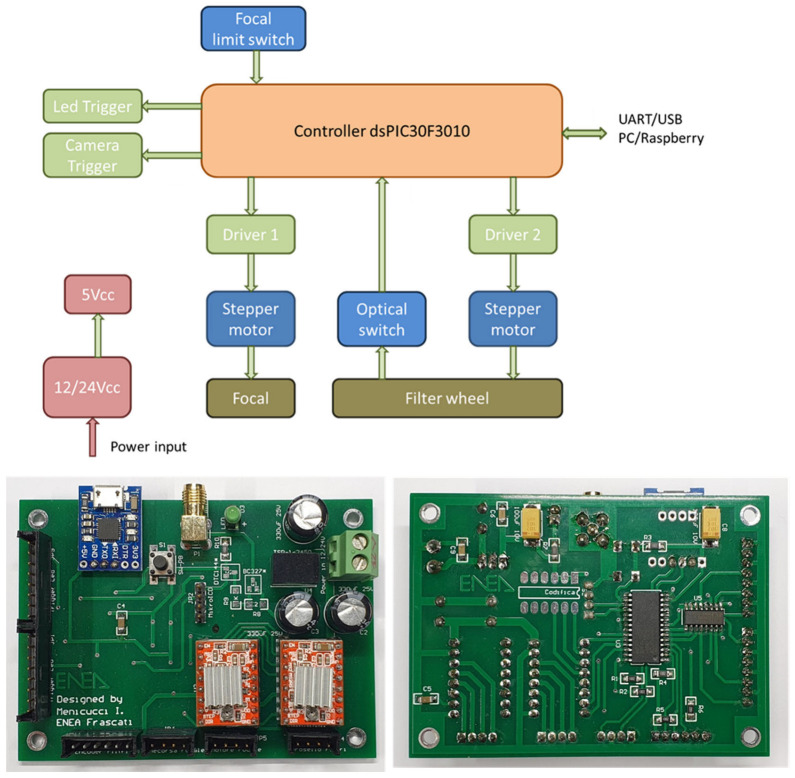
Schematic representation of the CLI’s control system and photos of the developed electronic board.

Some of the CLI components are driven by custom electronics developed in our laboratory, as shown in Figure 4. The electronic card is based on dsPIC30F3010 controller and is powered by 12–24 VDC. It allows synchronizing the LED light source (one per time) with the CMOS camera detector where the interval between the two triggers can be adjusted to compensate the inherent delays in the device activation. The camera images are locally acquired by a small single-board computer and sent via Wi-Fi or Ethernet to the laptop of the operator. The electronic board also controls the stepper motor of the LED’s focusing optics where the slit is brought to the zero position after the entire CLI system is turned on; successively, the position of the collimating lens is determined by relative step movements. The filter wheel is driven by the same electronic board: an optical switch is exploited to determine the zero-wheel position, while a filter is selected according to its angular distance from the switch. 

#### 2.1.5. Mobile Positioning System (MPS)

**Figure 5:**
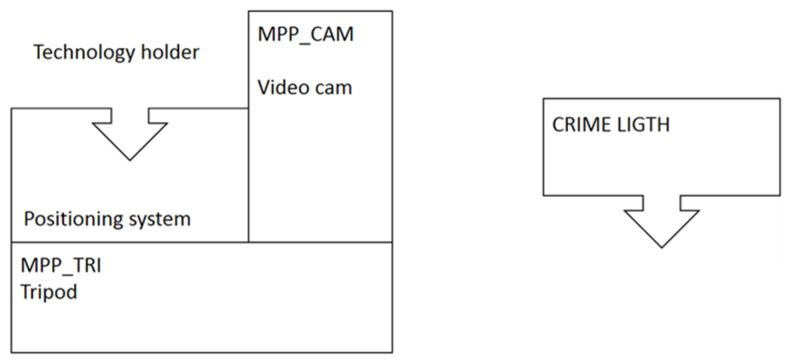
The MPS concept: the positioning system (**left**) houses different technologies (**right**) and provides the scene exploring, target pointing and scanning.

The MPS was designed to host any physically compatible stand-off sensor (Figure 5) in order to pass from a fixed-point measurement to a scene exploring and scanning. This system is remotely controlled, and it also provides inputs to position the area analyzed by a sensor in a wide geo-spatial context, as required for 3D scene reconstruction. The MPS is based on a robust tripod (Ibis Monolith) that bears a motorized platform (RAINBOW ASTRO RST135) with a plate containing a wide-angle color IP camera (frontal) and precise fast clumps for mounting of a sensor with a proper mechanical interface. The tripod made of carbonate fibers is lightweight (<3 kg), and itself can bear loads up to 50 kg. The motorized stage can be theoretically oriented in any direction throughout the solid angle by varying the angular position of two rotation actuators with mutually orthogonal axes. The platform rotations can be remotely operated via Wi-Fi or a serial link. The pre-aligned frontal panoramic camera provides live images that can be exploited for scene exploring and photo fragmentation. Once the central part of the scene to be analyzed by the sensor is located, whose measurement area is much more restricted compared to that visualized by the panoramic camera, the successive relative target locations probed by the sensor can be recovered from the motor positions with accuracy better than 1 arcsec (0.005 mrad). In the case of the CLI sensor, the initial sensor pointing on target is visualized by the red laser beam of the distancemeter and can be additionally improved by color images from the HD camera that has high spatial resolution (see Table 2). In addition to the remote operation of the MPS, the target pointing can be operated manually by the handheld HUBO controller; the last possibility is particularly advantageous for carrying out coarse positioning after the first placing of the instrument on a scene or after moving the tripod to another position.

Regarding the deployment of the CLI mounted on MPS, the system must be placed at a distance between 2 m and 10 m from the target. For each new physical position of the MPS, it is necessary to take and store the photographic and motor angle’s references, which are further exploited as inputs for accurate geo-localization and for eventual repeating of the sensor’s measurements at various target locations.

### 2.2. Software and Graphical User Interface (GUI)

**Figure 6:**
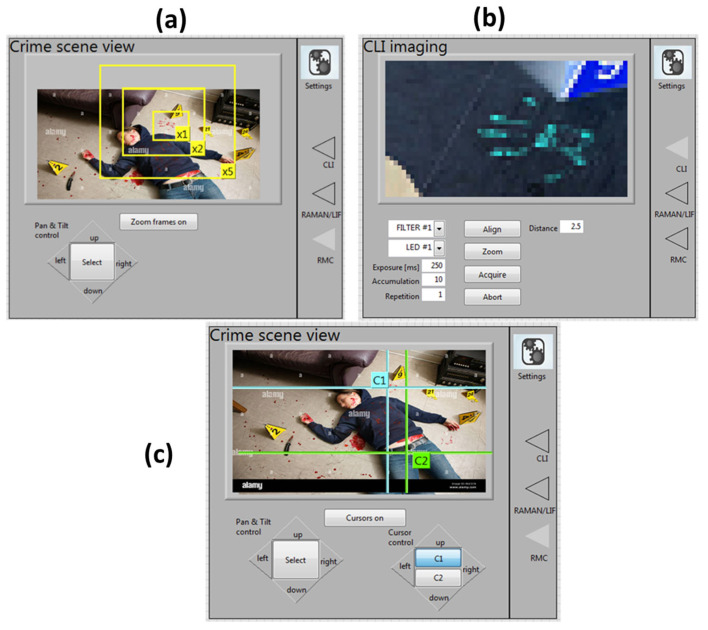
(**a**) The default zoom operation mode of the MPS. (**b**) Example of a high magnification central image taken by the CLI. (**c**) The view mode for scanning measurements inside the area selected by the cursors.

The software design features an intuitive graphical user interface that includes both the MPS and CLI controls via a computer desktop. The GUI development here was based on our previously developed Integrated Laser Sensor (ILS) for stand-off target scanning by Raman, Laser-Induced Fluorescence (LIF) and Laser-Induced Breakdown Spectroscopy (LIBS) techniques [39]. The GUI home window displays the live scene viewed by the panoramic camera mounted on the MPS. The scene can be explored by moving the two axis motors via four buttons available on the control panel (Figure 6a). A particular area of the scene could be zoomed up to five times to search for details. At a fixed position of the MPS, the central view of the target could be switched to the CLI mode that supplies a highly magnified image (Figure 6b). After taking the references for the central image position (see the previous subsection), the main control panel can also be switched to scanning mode, where the area to be examined by raster measurements is delimited by two interactively moving cursors C1 and C2 (Figure 6c).

**Figure 7:**
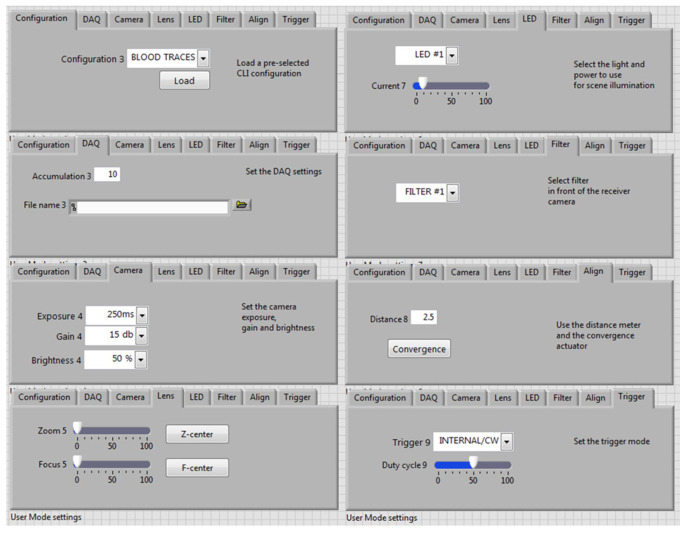
View of the User Mode settings tab.

The user interface implements a configuration panel that allows the CLI to be operated in a standard User Mode or in Expert Mode. The User Mode is accessible without a password and does not require specific training for the operator who runs field measurements. The operator can recall the pre-selected optimized settings for specific measurement scenarios (e.g., traces of biological fluids, chemical agents, etc.) but also has access to the menu (Figure 7), where some basic settings of the instrument can be modified. In this way, the operator has the possibility to finely tune and optimize a single measurement, thus maximizing the sensor performance for the specific target (trace/substrate combination) and ambient illumination. On the other hand, the Expert Mode for instrument operation has limited access to specifically trained operators, where additional instrument settings can be changed, including image resolution and rotation, data cropping, introduction of not standard filters, saving of default configurations, etc.

### 2.3. Operating the CLI in Field

**Figure 8:**
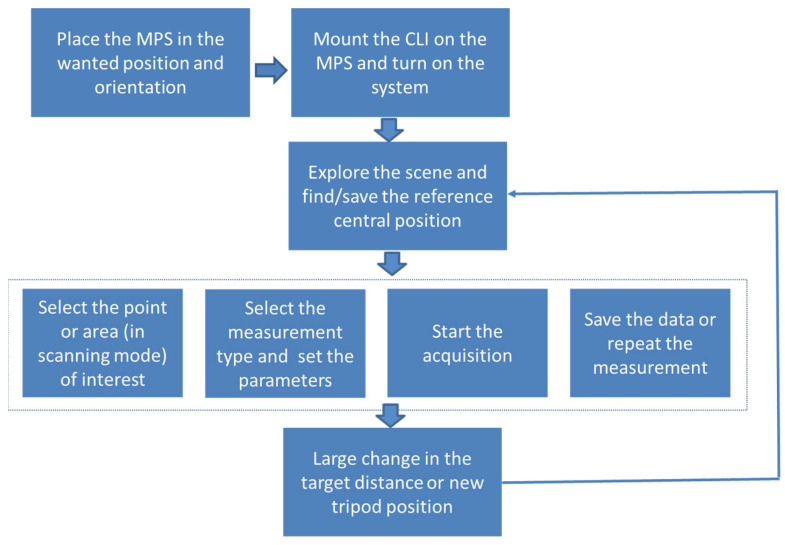
Flow chart for the CLI deployment and operation in field.

The CLI was conceived to be operated by one person, while two people may be needed to mount it on the mobile positioning system or to move the tripod with instrument to another position. The actual weights of the instrument head and box are 10 kg and 6 kg, respectively, and their physical separation facilitates the handling. The standard deployment and operating procedure are outlined in Figure 8. After installing the sensor head on the scanning platform and fixing the instrument box on the tripod or placing it nearby, the combined CLI/MPS system is turned on, and the control software is launched. When the ‘READY’ message appears on the GUI, it becomes possible to move the motorized platform and explore the crime scene: a live image from the panoramic camera appears on the monitor. The camera image can be zoomed to observe the details.

After selecting the wide area where measurements will be run at some points, we suggest switching to the target visualization by the CLI camera without filters and with a low objective zoom. At this point, the convergence actuator should be activated and the pointing light of the distancemeter should be brought to the center of the CLI’s image. Then, the reference position data, which include the images from the panoramic and CLI cameras, target distance and angular positions of the two axial motors of the MPS, should be saved. These data can be exploited for further 3D image reconstruction. Currently, the CLI/MPS system does not contain an automatic convergence finder; thus, if the target distance changed considerably from one measurement point to another, it is opportune to repeat the centering in order to take full advantage of the LED illumination of the target. 

From the main control panel, it is possible to select a single-point (small area) measurement or a rectangular region of interest to scan. In scanning mode, the default dimensions of the scan cells are automatically retrieved from the measured target distance and set focus of the CLI camera objective. Inside the selected area to scan, these cells will appear superimposed over the target’s image seen by the panoramic camera. 

In following, it is necessary to select the desired measurements (LED’s wavelengths and scattering and/or fluorescence mode) and modify the acquisition parameters (LED power and duty cycle, camera’s exposure, number of accumulated frames, triggering mode, dimensions of the acquisition cell) if they are different from the default ones. These parameters were set as default, but they could be modified by the operator inside the allowed limits. The measurements are executed in a pre-defined sequence by activating one LED-filter combination per time. Alternatively, it is possible to choose one type of measurement at a time without modifying the target area selection. 

Each measurement can be confirmed and saved before stepping to the next one; otherwise, it can be repeated. The operator may add an annotation for each image/data file. After the end of the selected measurement, as long as the scanning platform is not moved by the operator to another area of the scene, it is possible to repeat the measurement(s) exactly on the same area or on single cells of the scanning grid. 

The same procedure can be followed both for indoor and outdoor scenarios. The single-point measurement by CLI on forensic samples may take from less than one second to tens of seconds, depending on distance, background light and the response of the trace material and of the substrate. 

The present CLI prototype was realized starting from the inputs received from the end users, and it satisfied the initial requirements reported in Section 2.1. The prototype will be subjected to further improvements after the demonstration campaigns scheduled in the RISEN project. 

## 3. Results of Preliminary Testing of CLI

The first tests of the CLI sensor were performed on two classes of representative samples, namely fingerprints, the identification of which requires high-resolution images, and bodily fluids on substrates that make their observation difficult by visual inspection. Although we tested different kinds of fingerprints, we report here only the results obtained with apocrine secretions because they had the sharpest contours, which are important for evaluating the imaging performance of the CLI instrument. As representative cases for the detection of bodily fluids, we considered blood traces on black cotton and semen on white cotton. The details about the testing samples, measuring conditions and obtained results are given in the following subsections.

### 3.1. Sample Preparation and Testing Conditions

Groomed fingerprints containing apocrine secretions were collected in the following way: the donor’s hands were washed with soap, and fingertips were rubbed across the forehead to saturate the fingertips with sebaceous secretions. The fingertips from both hands were rubbed across one another to homogenize distribution of the groomed secretions among all 10 fingertips. The transfer on a silica wafer covered by an SiO_2_ layer (285 nm thick) was produced using an in-house developed fingerprint sampler that contained a lever-mechanism that assured a fixed amount of force (i.e., weight applied at the end of the lever). Using the handle of the lever, the substrate could be easily applied or removed from the finger for a selected duration (i.e., time of transfer). Here, the time of transfer was set to 5 s, while the weight of the lever was of 1 kg. 

Blood and semen samples were anonymously donated by healthy humans. The blood was collected by venipuncture into EDTA tubes in a clinic, and it tested negative to HIV and B and C hepatitis. Semen was self-collected by the donors into appropriate tubes. All the donations are approved by the University of Alcalá Ethic Committee of Research and Animal Experimentation under the codes CEIP/HU/2021/1/002 and CEIP/2022/3/045. The not-diluted blood was delivered in volume of 10 μL or 1 μL to a black cotton substrate, while 15 μL of semen was placed on a white cotton material. The stains were left to dry inside a flow cabinet for 24 h before measurement. 

Here, the reported CLI testing was performed by collimated LED’s illumination, corresponding the spot area on target of about 50 mm diameter. The LEDs were operated in continuous mode. 

The fingerprints were photographed at a distance of 7 m under 1 mW illumination by LED at 455 nm or 525 nm; the corresponding exposure times were 150 ms and 1000 ms, respectively. In case of the green light illumination, a cut-off filter at 530 nm was inserted in front of the camera. The comparative measurements under ambient light were also performed.

Blood stains were examined at distances of 5 m and 7 m under 2 mW LED emission at 850 nm, and the exposure time was 2 s. The stain of semen placed at distance of 7 m was photographed with 1 mW emitting LED at 385 nm, without and with a cut-off filter at 530 nm; the image acquisition took 1 s. 

### 3.2. Detection of Fingerprints by CLI 

**Figure 9:**
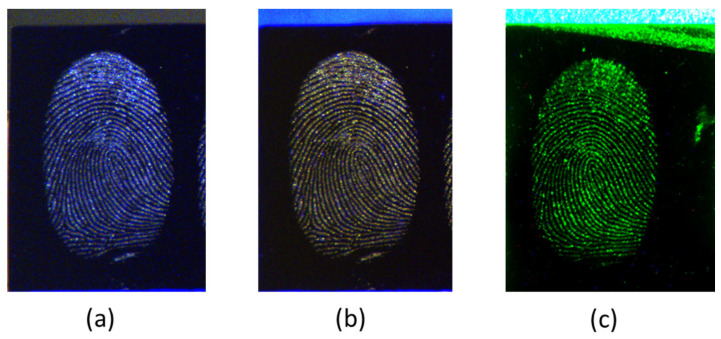
Photos of the apocrine fingerprint on the Si wafer taken by the CLI at a distance of 7 m under ambient illumination (**a**), the LED light at 455 nm (**b**), or LED light at 525 nm with a long-pass filter at 530 nm (**c**).

Photographs of the fingerprints on the Si wafer taken at working distance of 7 m confirmed a good spatial resolution of the images, which was also sufficient for the identification of a person. The Si wafer itself was highly absorbing for visible light, thus enhancing the contrast of the fingerprints without additional LED illumination (Figure 9a). In the presence of the LED light at 455 nm, a yellowish fluorescence of the apocrine fingerprint occurred (Figure 9b), while the elastic scattering of some tiny particles was also observed. The image of fingerprint taken under green LED light, where we had a combined effect of scattering and fluorescence above 530 nm, is shown in Figure 9c. Here, both the rims and some particles on the wafer were well visible.

**Figure 10:**
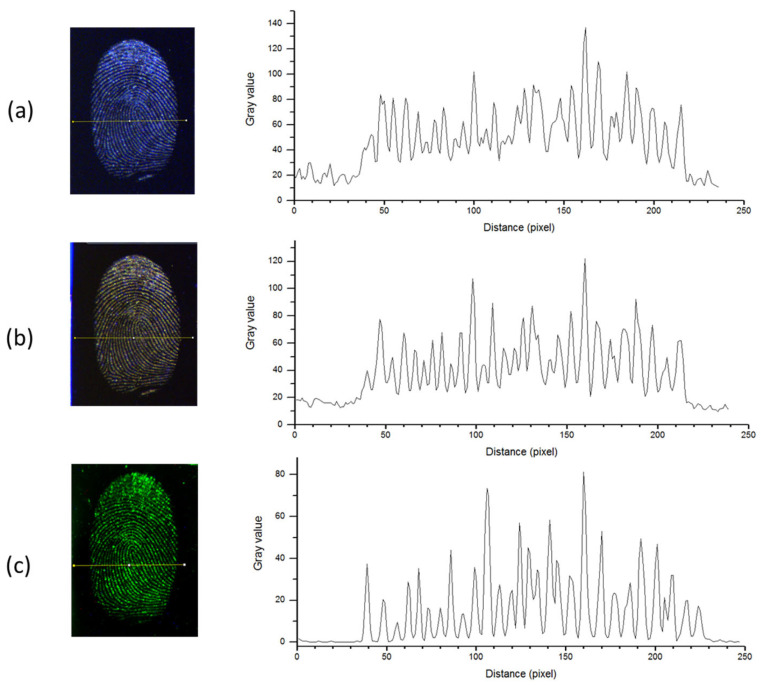
Selected line (in yellow) for the intensity distribution analysis of the apocrine fingerprints on the Si wafer under illumination by: (**a**) ambient light; (**b**) LED at 455 nm; (**c**) LED at 525 nm with a cut-off filter at 530 nm. The corresponding gray intensity pixel’s distributions are depicted in the right column.

In order to evaluate the image quality of the fingerprint photographs taken by CLI, we examined the equivalent gray intensity distribution along a line in the photographs (Figure 10(left)). The image analyses were performed by ImageJ software (1.54b, free license), and the results are shown in the right column of the Figure 10. For all three illuminations, the ridges were clearly visible from the alternation of peaks (P) and valleys (V) along the examined line. The baseline that represents noise was significantly higher for the image taken under ambient light than under LED illumination. The noise, calculated as the standard deviation over the first 30 pixels in the line, had the values of 4.83, 1.95 and 0.40 for ambient light, blue and green LED illumination, respectively. The largest difference between a peak and valley occurred around the pixel number 160. The contrast was calculated as C = log_10_(P/V) [40], where P is the peak value, and V is the minimum nearby pixel intensity. The resulting contrast for the selected peak was 0.615 and 0.785 for ambient and blue LED illumination, respectively. In the case of the green LED illumination, the contrast regarding the same peak/valley position was as high as 1.90. Both from the background noise and contrast, it is evident that the blue LED illumination already improved the image quality of the fingerprint compared to ambient light, and that this quality was further and drastically enhanced when detecting fluorescence induced by green LED light. The image resolution of the developed CLI system was sufficient for fingerprint identification while the relatively wide ridge peaks, involving 7–8 pixels, leave a possibility for various post-processing methods aimed to increase further the image quality [41].

### 3.3. Detection of Bodily Fluids by CLI 

**Figure 11:**
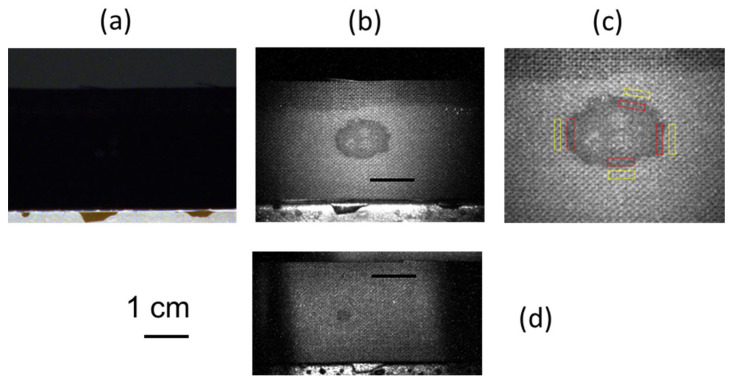
Photos of 10 µL of blood trace on black cotton taken at a distance of 5 m under: (**a**) environmental illumination; (**b**) LED light at 850 nm. (**c**) The areas selected for calculating the contrast. (**d**) Photo of 1 µL of blood trace on black cotton taken at a distance of 7 m under LED light at 850 nm.

The 10 µL blood traces on black cotton substrate could not be detected under environmental illumination even by the HR imaging provided by CLI, as shown in Figure 11a. However, the illumination by LED at 850 nm clearly indicated the presence of the trace (Figure 11b) thanks to the NIR absorption of blood [20]. The blood stain was still detectable at a distance of 7 m, where it was also possible to distinguish a stain from a blood droplet of only 1 µL volume (Figure 11d). The photograph of 10 µL of dried blood on black cotton under the LED illumination indicates the presence of a coffee ring effect, with the largest change in pixel intensity at the stain’s edges compared to the bare substrate. Here, the cotton substrate presented a knot structure with a period corresponding to about 5 pixels, which itself produced large variations in the image intensities. In the case depicted in Figure 11b, the gray pixel intensity across the bare substrate had an excursion of 80 counts on the 0–255 scale. In order to evaluate the contrast achieved at the stain’s edges, we calculated the mean pixel intensity, I_R_, at selected edge sections of the residue and the mean pixel intensity, I_B_, for the nearby background substrate with the same area. These measurements were performed at four places per picture, as illustrated in Figure 11c, and the average contrast C_av_ at edges of the blood residue was calculated as:

C_av_ = ∑(*i* = 1 ÷ 4) log_10_ (I*^i^*_B_/I*^i^*_R_) (1)

The stain from 10 µL of blood occupied an area of about 90 mm^2^, with an average contrast C_av_ of 0.149, calculated from Equation (1). The smallest photographed blood droplet, of 1 µL in volume, occupied an area of about 9 mm^2^. In this case, the value of I_R_ in Equation (1) was taken as an average over the residue; the calculated average contrast was 0.163, slightly higher than that for the larger blood droplet due to a less pronounced coffee ring effect.

The capability of CLI to detect a stain from only 1 µL of blood left on black cotton substrate at a distance of 7 m represents an important step toward the stand-off analysis of crime scenes considering that the existing instrument prototypes have failed to detect much larger blood stains on a dark substrate [37] when working at closer distances (up to 3 m) than CLI.

**Figure 12:**
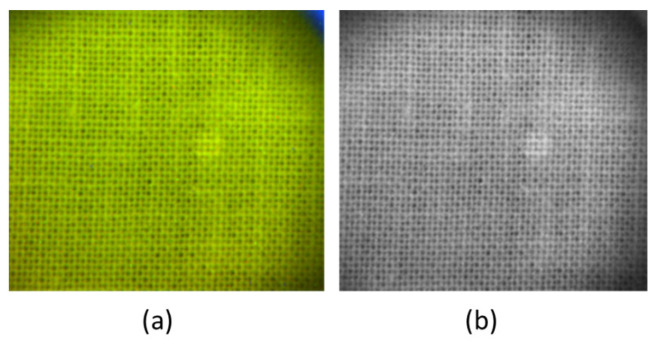
Photos of residue from 15 µL of semen on white cotton taken at a distance of 7 m under illumination by LED at 385 nm: (**a**) in color mode; (**b**) in B&W mode with a long-pass filter at 530 nm.

Semen on white cotton was not visible under ambient light, while LED illumination at 385 nm induced fluorescence of the substrate (Figure 12a). According to the literature [22], semen produces a broad fluorescence under near UV excitation, so the sample was further photographed after placing a long-pass filter (cut-off at 530 nm) before the CMOS camera (Figure 12b).

The 15 µL stain of semen occupied an area of about 10.4 mm^2^. The knot texture of the substrate had a period of about 0.63 mm in which the grey pixel intensity had very large excursions, up to 110 counts. The average contrast was calculated by taking into account the mean grey pixel value over the residue (I_R_) and the same over four nearby rectangular areas relative to the background (I_B_). Unlike the blood stains, the photographed trace of semen had a lighter color than the background fabric, and the contrast was retrieved from the modified Equation (1) as:

C_av_ = ∑(*i* = 1 ÷ 4) log_10_ (I*^i^*_R_/I*^i^*_B_) (2)

In this way, the resulting contrast of the trace on the white substrate was of 0.051 for the image taken under UV LED (Figure 12a). The contrast was slightly improved to the value of 0.056 after inserting the cut-off filter at 530 nm before the camera, which reduced the contribution of the fluorescence from the substrate.

Distinguishing 15 µL of semen on white cotton from a distance of 7 m from the CLI instrument can be compared to the results obtained in [36,37] for working distances up to 3 m. In the cited works, the detection rate of 100% and 80% was achieved for the sample volume of 250 µL and 5 µL, respectively.

## 4. Conclusions

In this work, we presented a novel stand-off CLI sensor capable of providing high-resolution color images of a target under ambient light or directed illumination from four high-power LEDs. A proper combination of the LEDs’ wavelength and filters in front of the detection camera opens the way to visualize various latent traces, directly or after sample treatment by standard fluorescent agents, as already well established in proxy scene examination by forensic lights. The CLI sensor is projected to operate at distances from 2 m to 10 m, and it can be used as a stand-alone device or integrated into the dedicated mobile positioning platform, as described in this work. This motorized platform has a high positioning accuracy that, together with the integrated panoramic camera, CLI camera and target pointing by laser beam of the distancemeter, provide all the necessary information for the successive accurate 3D scene reconstruction. The mobile positioning was integrated into the CLI’s control software, enabling scene exploration, target scanning and a precise selection of the points where to repeat or make new measurements. The whole measurement system can be battery-powered and operated remotely.

Preliminary testing of the sensor showed that the spatial photographic resolution at a target distance of 7 m was sufficient to perfectly resolve fingerprint ridges, while their contrast was drastically enhanced when detecting the fluorescence induced by green LED. Traces of blood on black cotton, invisible under ambient light, were captured by CLI under the LED illumination at 850 nm and with excellent sensitivity, visualizing a blood stain as small as 1 µL at a distance of 7 m. At the same distance, under UV LED illumination, it was possible to detect a 15 µL trace of semen placed on white cotton.

The presented instrument is the first demonstration of a true stand-off crime light imaging system and paves the way for a completely new approach to forensic crime scene examination. The proposed system is characterized by a high versatility and a modular design where the LED sources and camera filters can be replaced to adapt to the specific application.

Future testing of the CLI sensor concerns extensive measurements on various samples and substrates that could be found at a crime scene, both in the laboratory and during scheduled field trials of the RISEN project. Regarding the device development, the planned activities will be mainly focused on the implementation of short-exposure image acquisitions (~10 µs) synchronized with the LED pulses in order to minimize the influence of ambient light on the measurements. This operating mode is already arranged from the hardware’s point of view, and it requires the implementation of the fast merging of multiple images and visualization of the merged frame on the instrument graphical interface. 

## Figures and Tables

**Table 1:** Selected LED wavelengths, traces expected to be detected and the examination form.

λ [nm]	Range	Possible Applications	Examination
385	UV	Fingerprints	Light scattering/fluorescence/absorption
		Fibers	Fluorescence
		Body fluids	Fluorescence
455	VIS	(Treated) Fingerprints	Light scattering/fluorescence
		Body fluids	Fluorescence/absorption
		Fibers	Fluorescence
		Drugs	Absorption
520	VIS	(Treated) Fingerprints	Fluorescence
		Body fluids	Fluorescence
		Fibers	Fluorescence
850	NIR	Blood	Absorption
		Questioned documents (inks)	Fluorescence

**Table 2:** The calculated minimum size of the photographed area and the corresponding spatial resolution at various target distances.

Target Distance (m)	Min. Object Area (mm^2^)	Spatial Resolution (µm)
3	35.5 × 42.5	17
4	49.7 × 59.5	24
5	63.9 × 76.5	31
6	78.1 × 93.5	38
7	92.3 × 110.5	45
8	106.5 × 127.5	52
9	120.7 × 144.5	59
10	134.5 × 161.5	66

## Data Availability

The data of the measurements that support the findings of this study are available from the corresponding author upon reasonable request.
